# Intracycle power distribution in a heterogeneous multi-compartmental mathematical model: possible links to strain and VILI

**DOI:** 10.1186/s40635-022-00447-6

**Published:** 2022-06-01

**Authors:** Philip S. Crooke, Luciano Gattinoni, Michael Michalik, John J. Marini

**Affiliations:** 1 Department of Mathematics, Vanderbilt University, Nashville, TN USA; 2 Department of Anesthesiology and Intensive Care, Gottingen University, Gottingen, Germany; 3 Department of Medicine, University of Minnesota, Minneapolis, St. Paul, MN USA; 4 Pulmonary and Critical Care Medicine, Regions Hospital, University of Minnesota, MS 11203B, 640 Jackson St., Minneapolis, St. Paul, MN 55101-2595 USA

**Keywords:** Mechanical ventilation, Ventilator-induced lung injury, VILI, Mechanical power, Stress, Strain, Multicompartment, Mathematical model, Ventilation mode, Flow pattern

## Abstract

**Background:**

Repeated expenditure of energy and its generation of damaging strain are required to injure the lung by ventilation (VILI). Mathematical modeling of passively inflated, single-compartment lungs with uniform parameters for resistance and compliance indicates that standard clinical modes (flow patterns) differ impressively with respect to the timing and intensity of energy delivery—the intracycle power (ICP) that determines parenchymal stress and strain. Although measures of elastic ICP may accurately characterize instantaneous rates of global energy delivery, how the ICP component delivered to a compartment affects the VILI-linked variable of strain is determined by compartmental mechanics, compartmental size and mode of gas delivery. We extended our one-compartment model of ICP to a multi-compartment setting that varied those characteristics.

**Main findings:**

The primary findings of this model/simulation indicate that: (1) the strain and strain rate experienced within a modeled compartment are nonlinear functions of delivered energy and power, respectively; (2) for a given combination of flow profile and tidal volume, resting compartmental volumes influence their resulting maximal strains in response to breath delivery; (3) flow profile is a key determinant of the maximal strain as well as maximal strain rate experienced within a multi-compartment lung. By implication, different clinician-selected flow profiles not only influence the timing of power delivery, but also spatially distribute the attendant strains of expansion among compartments with diverse mechanical properties. Importantly, the contours and magnitudes of the compartmental ICP, strain, and strain rate curves are not congruent; strain and strain rate do not necessarily follow the compartmental ICP, and the hierarchy of amplitudes among compartments for these variables may not coincide.

**Conclusions:**

Different flow patterns impact how strain and strain rate develop as compartmental volume crests to its final value. Notably, as inflation proceeds, strain rate may rise or fall even as total strain, a monotonic function of volume, steadily (and predictably) rises. Which flow pattern serves best to minimize the maximal strain rate and VILI risk experienced within any sector, therefore, may strongly depend on the nature and heterogeneity of the mechanical properties of the injured lung.

**Supplementary Information:**

The online version contains supplementary material available at 10.1186/s40635-022-00447-6.

## Take home message

Potentially damaging tidal energy and power distribute asymmetrically within the heterogeneous environment of the acutely injured lung. Clinician-selected flow profiles not only influence the timing of power delivery, but also spatially distribute the attendant strains of expansion.

**Supplementary Information:**

The online version contains supplementary material available at 10.1186/s40635-022-00447-6.

## Introduction

A primary goal in the ventilatory support of patients with the acute respiratory distress syndrome (ARDS) is to safely relieve excessive breathing efforts while accomplishing adequate gas exchange. Because repeated expenditure of energy and its generation of damaging strain are required to injure the lung by ventilation, attention has turned toward pinpointing causal factors linked to mechanical power—commonly defined for the clinical setting as the product of energy per cycle and respiratory frequency [1, 2]. In recent work, we have called attention to the potential roles of the clinician-selected inspiratory flow pattern and the alveolar stress threshold in the delivery and distribution of damaging energy to the parenchyma [3, 4]. Our mathematical modeling of passively inflated, single-compartment lungs with uniform parameters for resistance (R) and compliance (C) indicates that standard clinical modes (flow patterns) differ impressively with respect to the timing and intensity of energy delivery—the intracycle power (ICP) that determines parenchymal stress and strain [3, 4]. While such a simplified one-compartment analysis is conceptually useful, it has limited applicability to the mechanically nonuniform, acutely injured lung. We did not link ICP to strain or strain rate, which are essential drivers of damage to biologic and inanimate materials [5–7]. The aim of the current work is to mathematically model the spatial distribution of intracycle inflation power, strain, and strain rate within a mechanically heterogeneous, multi-compartment lung in response to the global flow profiles imposed by each of the modes of ventilation commonly used in mechanical support. We first describe the rationale and construction of a multi-compartment mathematical model of ICP, next link ICP to strain, and then illustrate how clinician-made selections of mode, PEEP, VT and peak flow influence compartmental maximal strains and rates of strain. While still only concept-generating and highly simplified, doing so represents a further step toward developing a realistic predictive model of the alveolar strain and strain rate distributions characteristic of ventilated patients with ARDS.

## Methods

**Fig. 1:**
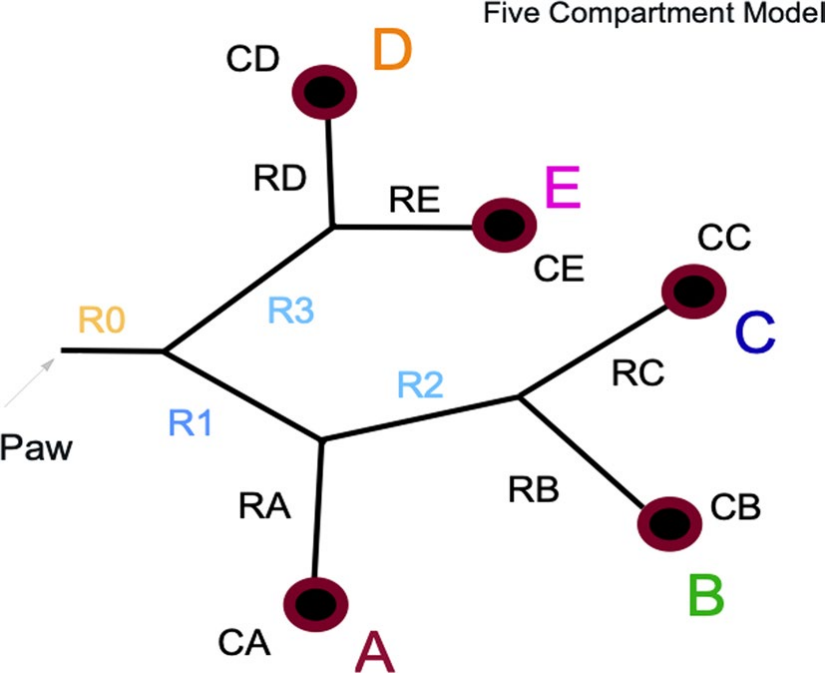
Schematic diagram of the 5-compartment model. Proximal shared resistances (R) are designated numerically; terminal, compartment-relevant resistances (R) and compliances (C) are designated alphabetically

The inflation energy per cycle includes the ‘elastic’ energy components that are conserved and stored at the alveolar level during breath delivery (termed ‘elastic’ energy) and the non-conserved energy component that dissipates during the inflation process (‘flow resistive’ energy) [8, 9]. The elastic component of ICP can be characterized as the elastic power that includes PEEP (termed ‘total elastic power’) and that element which selectively considers only incremental elastic pressure (‘driving power’) [9]. Given that our focus is on parenchymal injury, we consider 3 components of ICP (Driving, Elastic, and Total) during passive, controlled ventilation. Because repeated excessive strain and the rate of its development are believed to be key drivers of injury, we also relate our ICP metrics to analogs of strain and strain rate. Intracycle power and strain metrics for each of five compartments with varied mechanical properties were modeled for the ventilating modes currently prevalent in the clinical setting: constant flow (CF), regulated decelerating flow (DF, triangle, max  to 0 flow), and pressure targeted (‘pressure control’, CP) ventilation, as well as the sinusoidal flow pattern typical of spontaneous breathing (SF). In actuality, the micromechanics of heterogeneous lungs have numerous local variations conditioned by their immediate environments [10, 11]. At the micro-level, unit compliance is determined by the surrounding interstitial pressure, innate tissue pliability, reluctance of contiguous neighboring units to expand, and the rigidity of the local chest wall or pleural liquid that borders the alveolus in question. While recognizing this overwhelming biological complexity, in our model we choose to assign fixed compartmental values of R and C for simplicity and clarity of illustration (Fig. 1).

### Construction of the mathematical model

A mathematical model for the 5-compartment lung system was constructed using pressure and flow balances. (The mathematical details are provided in the Additional file 1). The model incorporates diverse compartmental resistances and compliances along with the resistances of the rigid connectors of those compartments to the airway opening (Table 1). The mathematical model is a system of linear differential equations for the compartmental volumes during inspiration $\left(0\le t\le {t}_{i}\right)$ and expiration $\left({t}_{i}\le t\le {t}_{\mathrm{tot}}\right)$ that allows pressure-controlled or flow-controlled ventilation at the airway opening. The solutions of the model yield dynamic compartmental volumes and flows during inspiration and expiration, along with end-expiratory (residual) pressures.

**Table 1:** Table of parameter values used throughout simulations unless otherwise stated

Compartment	A	B	C	D	E
Compartment resistance (cm H_2_O·s/l)	1	15	11	15	7
Compartment compliance (l/cm H_2_O)	0.08	0.06	0.05	0.02	0.02
Airway	R0	R1	R2	R3	
Resistances (cm H_2_O·s/l)	1	3	2	1	

### Instantaneous compartmental volumes and flows

**Fig. 2:**
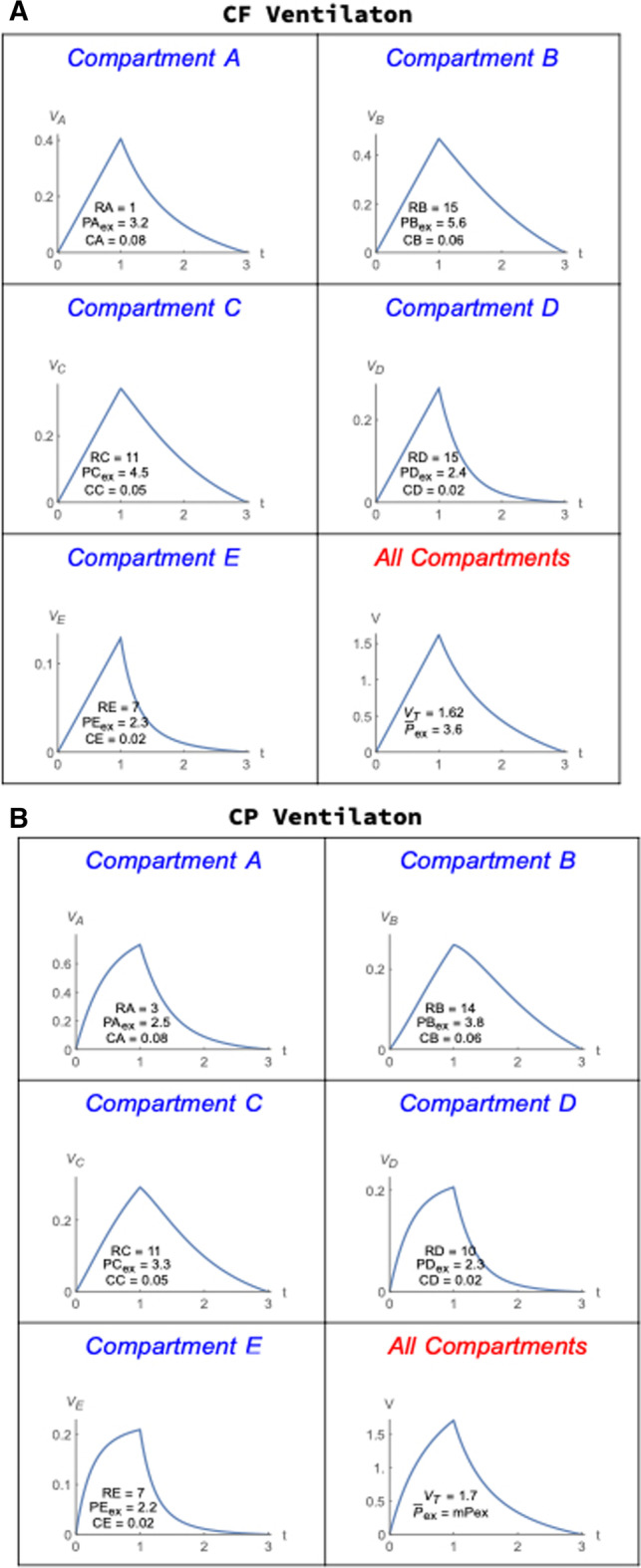
Compartmental volumes for a single complete tidal cycle of the same volume and inspiratory times during constant inspiratory flow (CF, **A**) and constant inspiratory pressure (CP, **B**). Note the variations among compartments in end-inspiratory volumes, accompanied by variations in deflation curvature

Two key purposes of the model are: (1) to determine how the intracycle power ($\mathrm{ICP}$) and flows distribute within the multi-compartment lung and (2) how those distributions impose strain on each compartment. Central to the calculation of these quantities are the instantaneous volumes and flows for each compartment under different modes of ventilation (Fig. 2). Clearly, innumerable combinations of compartmental resistances and compliances exist. For descriptive and comparative simplicity, we select a single set. Here is a summary of those parameters used in the multi-compartment simulations that will be described to follow: Shared piping resistances and variables are: $R0=1, R1=1, R2=2, R3=1.$ PEEP = 2 cmH_2_O; *t*_*i*_ = 1, *t*_tot_ = 3 s. The compartmental resistances, compliances and end-expiratory pressures are provided on the illustrated graphs of the volume profiles. The unit for resistance is $\mathrm{cm }{\mathrm{H}}_{2}\mathrm{O}\bullet \mathrm{s}/\mathrm{l}$; for compliance the unit is $\mathrm{l}/{\mathrm{cmH}}_{2}\mathrm{O}$; and for pressure, the unit is ${\mathrm{cmH}}_{2}\mathrm{O}$. For CF, we choose the flow at the airway opening as ${Q}_{aw}=1.7 \,\mathrm{l}/\mathrm{s}$ and for CP, ${P}_{\mathrm{set}}=13.75 {\mathrm{cmH}}_{2}\mathrm{O}$. To begin, compartmental volumes are partitioned as illustrated for CF and CP (Fig. 2).

### Distribution of intracycle elastic power

**Fig. 3:**
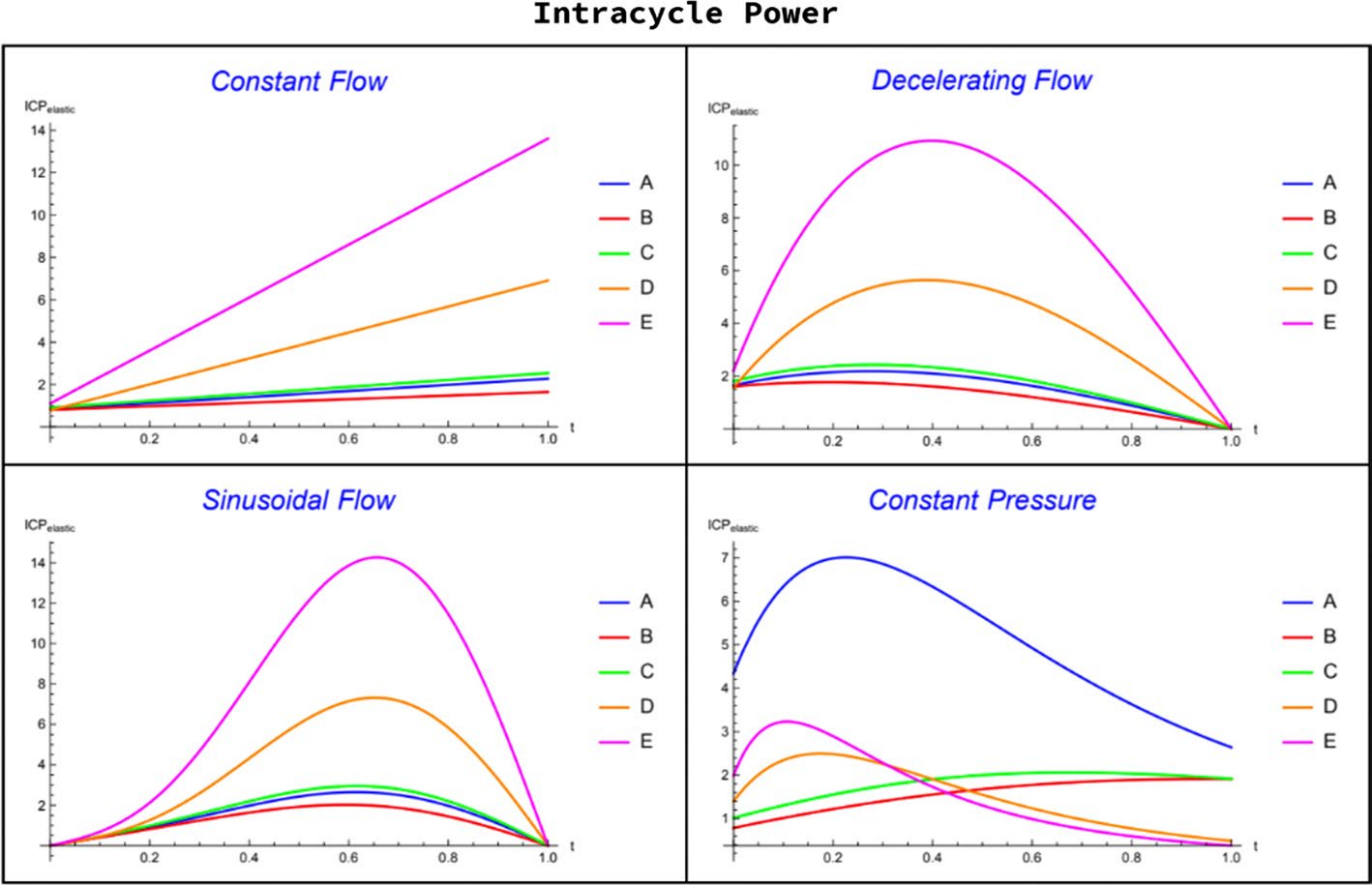
Comparison of inspiratory compartmental intracycle elastic power (ICP_elastic_) for all labeled modes of ventilation: constant inspiratory flow (CF), decelerating inspiratory flow (DF), constant inspiratory pressure (CP), sinusoidal inspiratory flow (SF)

Here we extend the concept of intracycle power (ICP) for a single-compartment model [2, 3] to this 5-compartment model. Our ultimate goal is to connect the intracycle power applied to each compartment to the strain dynamics experienced by that compartment during inflation. To consider tissue strain we focus on *total* intracycle elastic power $\left({\mathrm{ICP}}_{\mathrm{elastic}}\right),$ which we define mathematically as:

$${\mathrm{ICP}}_{\mathrm{elastic}}\left(t\right)=Q\left(t\right)\left(\frac{V\left(t\right)}{C}+{P}_{\mathrm{ex}}\right), 0\le t\le {t}_{i},$$

where $V(t)$ is the instantaneous volume in the compartment, $Q(t)$ is the instantaneous flow rate into the compartment, $C$ is the compartmental compliance, and ${P}_{\mathrm{ex}}$ is the residual pressure in the compartment at the end of expiration (total PEEP). The volumes, flows, and residual pressures are readily available from the mathematical model and depend on the mode of ventilation applied to the 5-compartment configuration (A through E). These compartments vary in their ranges of compliance and resistance, with A having the highest compliance, D the highest resistance, and the remaining compartments having mixed, intermediate values for resistance and compliance. Each compartment deflates passively at a rate largely governed by its own time constant. To illustrate how ${\mathrm{ICP}}_{\mathrm{elastic}}(t)$ varies from compartment to compartment and differs with mode of ventilation, we show this intercompartmental variation for three forms of controlled flow ventilation and for constant pressure ventilation, using the same parameters that were chosen for the prior compartment volume simulations (Fig. 3).

### Connecting intracycle power to strain for each compartment

To relate ICP to VILI, we propose a simple model that relates compartmental strain at time *t* (and strain rate, $\mathrm{strai}{n}^{^{\prime}}\left(t\right)$) to compartmental ICP. The model assumes that the compartments are inflatable uniform spheres with strain related to the ratio of volume at time *t* to rest (unstressed) volume at inflation onset (*V*_rest_). Note that in this macro-level model each of the five compartments is assigned the same initial space when unstressed, independently of the compliance parameter that characterizes it. We show in the Additional file 1 that the dynamic strain and the rate of strain at time *t* on the walls of the compartments are influenced by delivered elastic energy at time *t*, *A*(*t*), and by resting compartment volume subjected to zero pressure:

$$\mathrm{strain}\left(t\right)=1+\frac{\sqrt{2CA\left(t\right)+{C}^{2}{P}_{\mathrm{ex}}^{2}}}{ {V}_{\mathrm{rest}}},$$

$$\mathrm{strain}^{^{\prime}}\left(t\right)=\frac{\mathrm{ICP}(t)}{{V}_{\mathrm{rest}}\sqrt{{2CA\left(t\right)+C}^{2}{P}_{\mathrm{ex}}^{2}}},$$

where

$$A(t)=\underset{0}{\overset{t}{\int }}{\mathrm{ICP}}_{E}\left(s\right)\mathrm{ds}.$$

Here $C$ is the compartmental compliance, ${P}_{\mathrm{ex}}$ is end-expiratory pressure in the compartment, and ${\mathrm{ICP}}_{E}$ is the dynamic elastic intracycle power within the compartment. Strain is unitless, and strain rate has the dimension of 1/s.

## Modeling results

### Clinician-selected variables: mode, PEEP, VT and peak flow

#### Strain and strain rate as functions of time and mode

**Fig. 4:**
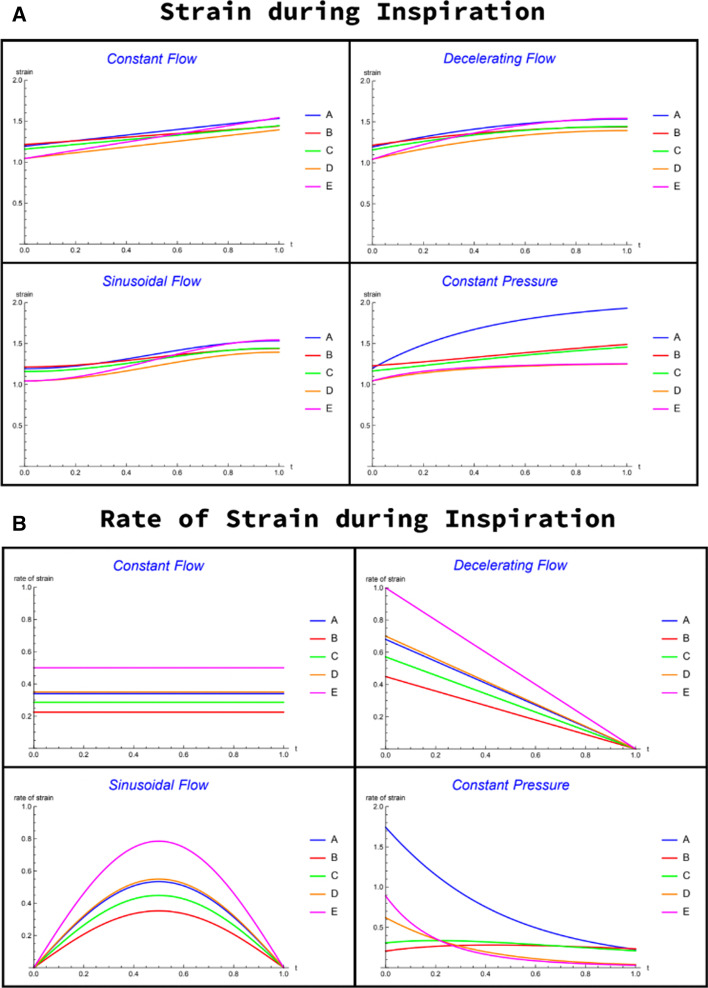
Compartmental strain (**A**) and strain rate (**B**) for all controlled flow and constant pressure ventilation modes. Note the marked differences among compartments, depending on flow profile

To illustrate the distributions of strain and rate of strain among compartments, we calculate these quantities for all modes (CF, DF, SF, PC) that we examined for compartmental volumes and intracycle elastic power (Fig. 4). For illustrative simplicity we arbitrarily assign the resting volume to be the same for each compartment: ${V}_{\mathrm{rest}}=1$. However, the model does allow different compartmental resting volumes (‘sizes’), as we discuss subsequently.

#### Maximum strain versus PEEP

In a previous section, we modeled strain developing as a function of ventilator-produced volume $V(t)$ such that $V\left(0\right)=0$, $V\left({t}_{i}\right)={V}_{T}$, and the end-expiratory pressure ${P}_{\mathrm{ex}}$ is (PEEP + auto-PEEP):

$$\mathrm{strain}\left(t\right)=1+\frac{V\left(t\right)+C{P}_{\mathrm{ex}}}{{V}_{\mathrm{rest}}}.$$

**Fig. 5:**
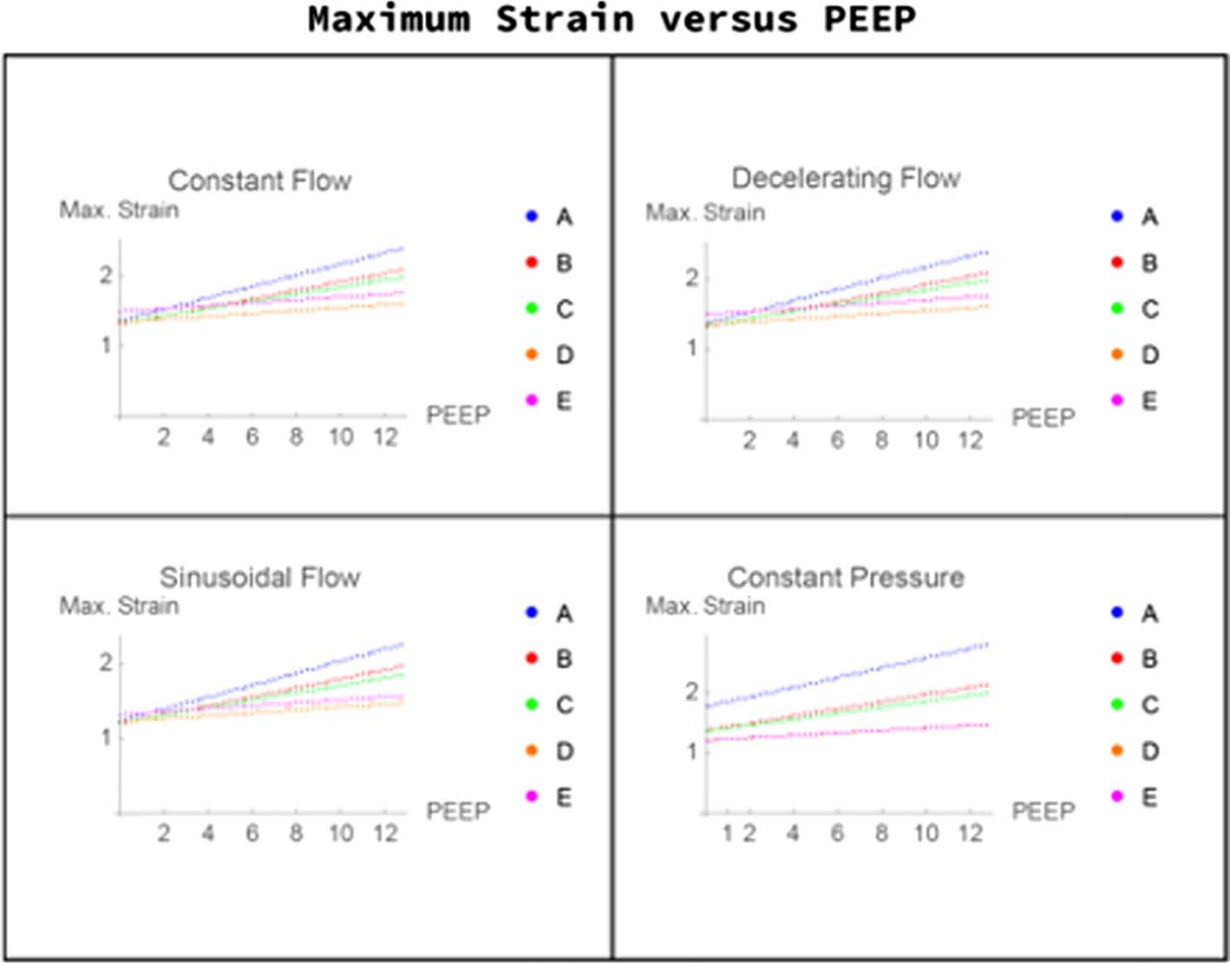
Maximum compartmental strains as a function of PEEP for the four modes of ventilation. PEEP causes maximal strains to rise monotonically but to different degrees in all compartments

We now investigate the effects of PEEP on the dynamic strain in each compartment. In particular, we calculate the level and rate of strain for each compartment for a spectrum of applied PEEPs: $0\le \mathrm{PEEP}\le 12.75 \mathrm{cm }{\mathrm{H}}_{2}\mathrm{O}$. We repeat these calculations for each of the four modes of ventilation using the same compartmental parameters and ventilator settings and plot the maximum strain for each compartment against PEEP settings (Fig. 5). As illustrated, while maximal strain predictably rises with PEEP in each compartment, its influence on maximal strain varies between them in all modes and distributes differently for pressure as opposed to flow-regulated ventilation.

#### Maximum strain versus VT and peak flow

In ventilating a mechanically heterogeneous system the clinician seeks settings, e.g., mode of ventilation, frequency, tidal volume, inspiratory time that achieve the ventilation goal while minimizing risk for damage to the lung. In the Additional file 1, we carry out in silico experiments over wide ranges of maximal flow and tidal volumes to determine which modes of ventilation minimize the maximum compartmental strain for different tidal volumes and maximum flow settings on the ventilator. The latter is of particular interest, as crossing a strain threshold is theoretically needed before energy and power become damaging [3, 4, 9]. As expected, strain rises monotonically with VT in each compartment. However, for DF and SF ventilation, we detect some nonlinearity in the behavior of the maximum strain as functions of set tidal volume and set maximum flow, rising more rapidly at higher tidal volumes for a specified ${Q}_{\mathrm{max}}$. This behavior is primarily due to the required increase in inspiratory time, which in turn increases the auto-PEEP, a component of the dynamic strain. Finally, there are variations in the maximum compartmental strains encountered with a given tidal volume according to which mode of ventilation is used.

### Modification of the model for heterogeneous compartmental rest volumes

In the model simulations that have been presented so far, we arbitrarily assigned the same resting volume $\left({V}_{\mathrm{rest}}\right)$ to each compartment $\left({V}_{\mathrm{rest}}=1\right)$. Recalling that the dynamic stain was modeled by the equation

$$\mathrm{strain}\left(t\right)=1+\frac{V\left(t\right)+C{P}_{\mathrm{ex}}}{{V}_{\mathrm{rest}}},$$

where $V\left(t\right)$ is the dynamic volume of the compartment above residual, $C$ is the compliance of the compartment, and ${P}_{\mathrm{ex}}$ is the end-expiratory pressure of the compartment, the compartmental maximum strain (${S}_{\mathrm{max}})$ is then

$${S}_{\mathrm{max}}=\mathrm{strain}\left({t}_{i}\right)=1+\frac{{V}_{T}+C{P}_{\mathrm{ex}}}{{V}_{\mathrm{rest}}}.$$

Choosing the same ${V}_{\mathrm{rest}}$ for each compartment was a convenient way to compare the compartmental maximum strains for different modes of ventilation with the same physiologic setup. We now relax this assumption of uniform compartmental rest volumes $\left({V}_{0}=1\right)$ to examine the effects of rest volume on the maximal strains.

**Fig. 6:**
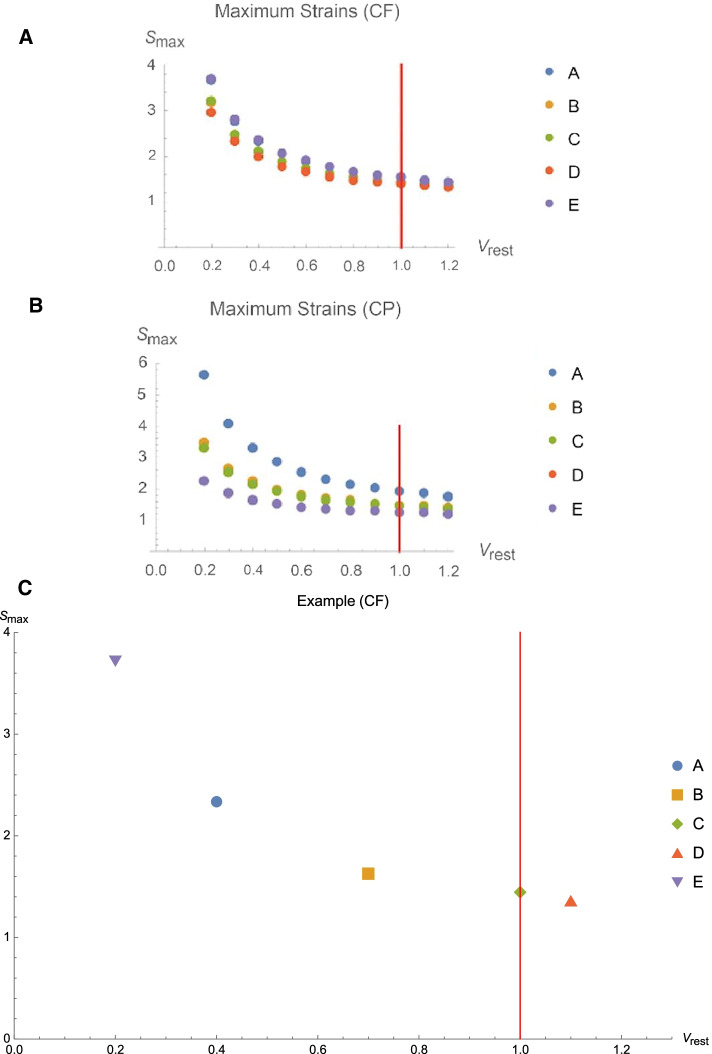
**A** Effect of resting volume (*V*_rest_) on maximal compartmental strains (*S*_max_) for CF (**A**) and CP (**B**). Calculated strains rise in all compartments as inverse functions of *V*_rest_. Variation of resting compartmental volumes at maximal strain during constant flow (**C**). Note that each compartment has a unique resting volume at which strain is maximized

Our first computational experiment is to assess how the compartmental maximum strains change as ${V}_{\mathrm{rest}}$ varies over the range, $0.2\le {V}_{\mathrm{rest}}\le 1.2$, in constant flow and constant pressure ventilation assigning the same physiologic (R & C) parameters used in the previous simulations (Fig. 6A and B).

As expected, all maximal compartmental strains increase as the rest volumes decrease. However, how much the maximal compartmental strains change as function of *V*_rest_ depends on the R & C parameters of the 5-compartment model and on the clinician-set ventilator parameters, as well. The same type of graph was created with decelerating flow ventilation and sinusoidal flow ventilation (not illustrated). There were very few qualitative or quantitative differences between them and Fig. 6A and B depicting CF and CP.

The above simulation can be adapted for calculating the distribution of maximal compartmental strains when compartments are assigned different rest volumes. For example, if we set different resting compartmental volumes for compartments with high (e.g., A) and low (e.g., D) compliances (Table 1), then the distribution of compartmental maximum strains varies, as well (Fig. 6C). In the illustrated case of CF ventilation, Compartment A encounters the highest maximal strain and Compartment D the lowest for these compartmental rest volumes, using our standard R and C parameter settings that are designated in Table 1.

## Discussion

The primary findings of this model/simulation indicate that: (1) the strain and strain rate experienced within a modeled compartment are nonlinear functions of delivered energy and power, respectively; (2) for a given combination of flow profile and tidal volume, resting compartmental volumes influence their resulting maximal strains in response to breath delivery; (3) flow profile is a key determinant of the maximal strain as well as maximal strain rate experienced within a multi-compartment lung. By implication, different clinician-selected flow profiles not only influence the timing of power delivery, but also spatially distribute the attendant strains of expansion among compartments with diverse mechanical properties.

In recent modeling we considered the lung as a single compartment with uniform resistance and compliance characteristics; nonetheless, the maximum ICP value and the energy delivered per breath above an arbitrarily designated pressure threshold (set for the entire lung) varied considerably with the selected flow profile [3, 4]. Our current work extends that line of investigation by showing how ICP and strain might distribute spatially over time in the mechanically heterogeneous environment of the acutely injured lung. To our knowledge this study represents the first attempt to develop and use a multi-compartment mathematical model focused on regional power and strain primed with clinically familiar input variables.

### Relationship of ICP to strain and strain rate

Although measures of elastic ICP may accurately characterize instantaneous rates of energy delivery, how the ICP component delivered to a compartment affects the VILI-linked variable of strain is determined by compartmental mechanics, compartmental size and mode of gas delivery. Importantly, the contours and magnitudes of the compartmental ICP, strain, and strain rate curves are not congruent; in other words, strain and strain rate do not necessarily follow the compartmental ICP, and the hierarchy of amplitudes among compartments for these variables may not coincide. For any given compartment, different flow patterns impact how strain and strain rate develop as compartmental volume crests to its final value. Notably, as inflation proceeds, strain rate may rise or fall even as total strain, a monotonic function of volume, steadily (and predictably) rises. Which flow pattern serves best to minimize the maximal strain rate experienced within any sector, therefore, may strongly depend on the nature and heterogeneity of the mechanical properties of the injured lung.

### Limitations

For clarity and simplicity of comparisons, we assumed non-contiguous, spheroid geometry as a starting point for our multi-compartment analyses, defining strain in terms of the incremental volume: $\mathrm{strain}(t)=1+\frac{V\left(t\right)+C{P}_{\mathrm{ex}}}{{V}_{\mathrm{rest}}}$*.* The stresses and resulting strains do not distribute uniformly within the non-spherical or irregularly shaped structures that are more characteristic of biologic alveolar tissues. The theoretical importance of geometry to the local strains that are experienced in response to identical lung unit distending pressures is illustrated in Additional file 1: Fig. S3. Another clear limitation in our exposition of this initial multi-compartment model is the unchanging parameters for R & C used to characterize regional properties of each compartment; again with the objective of clarity in mind, we did not vary the maximum range of those properties among compartments and no considerations were made of viscoelastance, stress risers [12, 13] or nonlinear behaviors (e.g., tidal opening and closure).

In our 5-compartment model we make no attempt to allow the expansion of one compartment to directly alter the functional compliance of contiguous others. But such modeled separation of compartments does have structural parallels within the injured lung. For example, the left lung is separated anatomically from the right lung, and fibrous fissures divide the lobes of each—five in total. Moreover, the most gravitationally dependent regions are not immediately contiguous with non-dependent zones. Thus, when considered on the whole lung and lobar scales, even the acutely injured lung comprised disconnected ‘zones’ with differing compliance characteristics that are served by a network of interactive airways that distribute total flow. The relative ease or difficulty of each compartment’s inflation does influence the flows and strains that occur elsewhere. Interacting via the branches of the network, the RC properties of each help determine the share of the total flow that they receive and the fraction apportioned to the others. For these reasons, our admittedly unrefined mathematical model should be viewed and interpreted on a macromechanical rather than a micromechanical level.

### Clinical implications

Assuming that inflation energy and intracycle power are linked to VILI, they are likely to do so through their effects on strain and strain rate [14, 15]. Indeed, biologic experiments strongly suggest a role for flow amplitude in the generation of VILI [16, 17]. As a relatively new direction in the investigation of VILI energetics, the relationship of global intracycle power to strain experienced by mechanically diverse compartments has not been defined. We reasoned that the elastic component(s) of ICP, i.e., those portions influenced by PEEP and driving pressure, should be the primary power components of concern. This multi-compartment mathematical model, which employed inputs familiar to the clinician such as R and C, demonstrates that the ICP values necessary to produce local damaging strain are influenced not only by the flows and pressures as applied and measured at the airway opening, but also by the range of mechanical properties of the units that comprise it.

Although grounded on strong physical principles, the quantitative results from these highly simplified simulations from a multi-compartment mathematical model clearly cannot be applied directly to the clinical setting. Indeed, it is intended for conceptual and not practical purposes. We did not seek to identify the optimized flow and power distributions that minimize strain or strain rate. A few messages, however, do seem immediately transferrable. Prominent among these are: (1) flow amplitude and profile, heretofore underemphasized as contributors to a lung protective strategy, significantly influence the distribution as well as the timing and magnitude of the maximal strain and strain rate imposed by a given tidal volume; (2) uniformity of transpulmonary pressures and resting volumes tends to reduce maximal compartmental strain; therefore, appropriate selections of body position and PEEP to encourage mechanical uniformity may help reduce VILI risk.

## Conclusion

The results of this multi-compartment model, though hardly definitive without further refinement, represent a step toward quantitatively defining the biomechanics of VILI generation.

## Supplementary Information

**Additional file 1.** Construction of the mathematical model and illustrated impact of geometry on intracompartmental strain.

## Data Availability

The granular datasets used and/or analyzed during the current study are available from the corresponding author upon reasonable request. All data generated or analyzed during this study are summarized in this published article and its additional information files.

## Abbreviations

ARDS: Acute respiratory distress syndrome
ICP: Intracycle power
ICP_elastic_: Intracycle power spent overcoming elastic pressures (PEEP plus tidal elastic pressure)
VT: Tidal volume
PEEP: Positive end-expiratory airway pressure
*P*_ex_: Total end-expiratory alveolar pressure (PEEP plus Auto PEEP)
R: Resistance
C: Compliance
*t*: Time since inflation onset
*t*_i_: Duration of inspiratory period
*t*_tot_: Total breath duration
Q: Flow
*Q*_max_: Maximum inspiratory ventilator flow
*P*_set_: Targeted inflation airway pressure above PEEP
*V*_rest_: Resting compartmental volume in absence of PEEP or inflation pressure
*A*(*t*): Elastic energy at time t
CF: Constant inspiratory airflow (mode)
CP: Constant inspiratory airway pressure (mode)
DF: Decelerating inspiratory airflow with triangular waveform (mode)
SF: Sinusoidal inspiratory airflow (mode)
*S*_max_: Maximal compartmental strain
