# Supplementary material for: Intracycle power distribution in a heterogeneous multi-compartmental mathematical model: possible links to strain and VILI
Source: Intensive Care Med Exp. 2022 Jun 1;10:21. doi: 10.1186/s40635-022-00447-6 (PMC9156592; doi:10.1186/s40635-022-00447-6)
Supplement: Supplementary file 1 — Additional file 1. Construction of the mathematical model and illustrated impact of geometry on intracompartmental strain. [file 40635_2022_447_MOESM1_ESM.docx]

***Supplement* for: Intracycle Power Distribution in a Heterogeneous Multi-compartmental Mathematical Model: Possible Links to Strain and VILI**

**Construction of Mathematical Model**

The mathematical model for the alveolar volumes, flows, and pressures are based on the following construction. The model is composed of five compartments (labelled A, B, C, D, and E) (Figure S1). The compartments are connected to the airway opening by inelastic pipes that have uniform resistances (R0, R1, R2, and R3). Each compartment has one connection to the piping network. The resistances to flow into and out of the compartments are denoted by the abbreviations: RA, RB, RC, RD, and RE. The compartments are expandable elastic spheres with compliance values: CA, CB, CC, CD, and CE.

**Figure S1:** Each compartment (A, B, C, D, E) is connected to the central airway by rigid segments that impede airflow (R0 --> R3). In addition, each subunit (Z) has a local combination of resistance R_Z_ and compliance C_Z_. Pressure at the airway opening is denoted by P_aw_. The fundamental premise of the model is that pressure at each junction point is the balance of those above (proximal to) and below (distal to) it.

| **Symbol** | **Description** |
| --- | --- |
| CA, CB, CC, CD, CE | Compartmental Compliances |
| RA, RB, RC, RD, RE | Compartmental Resistances |
| R0, R1, R2, R3 | Pre-Compartmental Airway Resistances |
| ${PA}_{ex}, {PB}_{ex}, {PC}_{ex}, {PD}_{ex}, {PE}_{ex}$ | Compartmental End-expiratory Pressures |
| $V_{A}, V_{B}, V_{C}, V_{D}, V_{E}$ | Dynamic Compartmental Volumes |
| ${VT}_{A}, {VT}_{B}, {VT}_{C}, {VT}_{D}, {VT}_{E}$ | Compartmental Tidal Volumes |

Two parts comprise the ventilation cycle: (1) inspiration $\left( 0\leq t\leq t_{i} \right)$ and (2) expiration $\left( t_{i}\leq t\leq t_{tot} \right)$. Here t is the time after inflation onset (0), and *t_i_ and t_tot_* designate the times at which inflation and deflation end, respectively. During inspiration, the ventilator delivers a regulated flow to the airway opening and during expiration, applies a prescribed constant pressure $\left( PEEP \right)$. The model provides a mathematical description of the volumes, pressures and flows that develop in each compartment over a single breath, enabling the computation of strain and strain rate (see following). Secondarily, it also determines flows within the piping network.

The model is constructed by assembling the pressures and flows at each node in the network. An example of a node is the three-limb juncture that connects the pipes with resistances $R1 \& R2$ with that of compartment $A,$ which has a compliance $CA$ and resistance $RA$. The dynamics of the compartment (flows and pressures) are described in our previous single compartment modeling paper that addresses intracycle power (Ref S1)**.** In particular, the pressure applied to a compartment will be of the form:

$$R\left( \frac{dV}{dt} \right)+\frac{V}{C}+P_{ex}=P_{node}$$

where $V(t)$ is instantaneous volume of the compartment, $R$ is the flow resistance of the compartment, $C$ is the compliance of the compartment, $P_{ex}$ is the residual pressure at the end of expiration in the compartment, and $P_{node}$ is the pressure at the node. This calculation is performed for each node in the network. The collection of equations yields a system of differential equations (one system for inspiration and one system for expiration) of the following form:

$$RA\frac{dV_{A}}{dt}=a_{1}V_{A}+a_{2}V_{B}+a_{3}V_{C}+a_{4}V_{D}+a_{5}V_{E}+P_{1}$$

$$RB\frac{dV_{B}}{dt}=b_{1}V_{A}+bV_{B}+b_{3}V_{C}+b_{4}V_{D}+bV_{E}+P_{2}$$

$$RC\frac{dV_{C}}{dt}=c_{1}V_{A}+c_{2}V_{B}+c_{3}V_{C}+c_{4}V_{D}+c_{5}V_{E}+P_{3}$$

$$RD\frac{dV_{D}}{dt}=d_{1}V_{A}+d_{2}V_{B}+d_{3}V_{C}+d_{4}V_{D}+5V_{E}+P_{4}$$

$$RE\frac{dV_{E}}{dt}=e_{1}V_{A}+e_{1}V_{B}+e_{3}V_{C}+e_{4}V_{D}+{ae}_{5}V_{E}+P_{5}$$

where $a_{i}, b_{i}, c_{i}, d_{i}, e_{i},P_{i}, i=1,2\ldots,5$ are constants that depend on the parameters of the system (compliances, resistances, and residual pressures). As noted, there exist one system of equations of this form for inspiration and one for expiration. Furthermore, in the case of flow-controlled modes of ventilation, each system of differential equations for inspiration reduces to a set of algebraic ones. Only in the case of pressure-controlled ventilation $\left( P_{aw}=P_{set} \right)$, are the modelling expressions two sets of differential equations. For pressure-controlled ventilation, the system of differential equations for inspiration has the initial conditions: $V_{A}\left( 0 \right)=V_{B}\left( 0 \right)=V_{C}\left( 0 \right)=V_{D}\left( 0 \right)=V_{E}\left( 0 \right)=0$. For all modes of ventilation, the system of differential equations for expiration has the initial conditions: $V_{A}\left( t_{i} \right)={VT}_{A},$ $V_{B}\left( t_{i} \right)={VT}_{B}, V_{C}\left( t_{i} \right)={VT}_{C}, V_{D}\left( t_{i} \right)={VT}_{D}, V_{E}\left( t_{i} \right)={VT}_{E}$ where ${VT}_{I}$are the tidal volume components distributed to individual compartments that sum to VT.

The residual pressures (PEEP + auto-PEEP) are denoted as ${PA}_{ex,} {PB}_{ex,}{PC}_{ex,}{PD}_{ex,}{PE}_{ex,}$ and are initially unknown, as are the tidal volumes that ultimately will distribute among the compartments. Once the solutions of the inspiratory and expiratory systems of equations have been found i.e., $V_{A}\left( t \right), V_{B}\left( t \right), V_{C}\left( t \right),$ $V_{D}\left( t \right), V_{E}\left( t \right)$ , then requiring $V_{I}\left( t_{i} \right)={VT}_{I}$ for inspiration and $V_{I}\left( t_{tot} \right)=0$ for expiration, $I=A,B,\ldots E$, yields systems of algebraic equations whose solutions yield the tidal volumes and residual pressures for each individual compartment.

**Connecting Intracycle Power to Strain for each Compartment**

A basic objective in our modeling of VILI is to connect intracycle power to the stress and strain experienced by individual compartments during the different flow profiles (modes) of ventilation. With this goal in mind and for clarity of illustration, we choose spherical elastic balloons as simple physical representations of individual compartments. The stress on the compartment is generated by its internal pressure, and the strain is the ratio of the volume of the inflating balloon to its initial (residual) volume before inflation begins (*V*_rest_). In terms of compartmental volume and elastic pressure, we define stress and strain as:

**Strain:** $strain=\frac{V\left( t \right)+CP_{ex}+V_{rest}}{V_{rest}}=1+\frac{V\left( t \right)+CP_{ex}}{V_{rest}}$

$$\Longrightarrow V\left( t \right)=V_{rest}\left( strain\left( t \right)-1 \right)-CP_{ex}$$

**Stress:** $stress\left( t \right)=P\left( t \right)=\frac{V(t)}{C}+P_{ex}$

$$=\frac{V_{rest}\left( strain\left( t \right)-1 \right)-CP_{ex}}{C}+P_{ex}=\frac{V_{rest}\left( strain\left( t \right)-1 \right)}{C}$$

Here, $V_{rest}$is the initial volume of the compartment before the tidal inflation, $V\left( t \right)$ is the *dynamic* volume of the compartment, so that at inflation onset, time 0, $V\left( 0 \right)=0$ and $V\left( t_{i} \right)=V_{T}$, and $P(t)$ is the dynamic elastic pressure in the compartment. We note that with these assumptions, the stress-strain relationship in this model is linear.

Recalling that the elastic intracycle power (*ICP_E_*) is defined as the product of the elastic pressure in the compartment with the flow into that compartment, in mathematical terms, we have:

$${ICP}_{E}(t)=pressure\times flow=\left( \frac{V(t)}{C}+P_{ex} \right)\frac{dV}{dt}$$

Therefore,

$${ICP}_{E}(t)=\left( \frac{V_{rest}\left( strain\left( t \right)-1 \right)-CP_{ex}}{C}+P_{ex} \right)\left( V_{rest}strain'(t) \right)$$

$$\Longrightarrow{ICP}_{E}\left( t \right)=\frac{{V_{rest}}^{2}}{2C}\frac{d}{dt}\left[ {strain}^{2}\left( t \right) \right]-\left( \frac{{V_{rest}}^{2}}{C} \right)strain'(t)$$

Here $strain^{'}\left( t \right)$ is the *rate* of change in strain as a function of the time of observation.

Integrating and assuming $strain\left( 0 \right)=1+\frac{CP_{ex}}{V_{rest}}$, we have:

$$\int_{0}^{t} ICP\left( s \right)ds=\frac{{V_{rest}}^{2}}{2C}\left[ {strain}^{2}\left( s \right)-2strain(s) \right]|_{s=0}^{s=t}$$

Solving this equation for $strain(t)$, we find:

$$strain\left( t \right)=1+\frac{\sqrt{2CA\left( t \right)+C^{2}P_{ex}^{2}}}{V_{rest}}$$

where

$$A(t)=\int_{0}^{t} {ICP}_{E}\left( s \right)ds$$

Notice that the $A(t)$ is the area under the intracycle elastic power curve at time t. Furthermore, we see that strain increases as the ICP increases. The strain function depends on the compartmental compliance and the end-expiratory pressure. In particular, $strain \sim\sqrt{A}$. This implies that the strain is proportional to the square root of the elastic energy. Furthermore, the rate of change in strain can be computed from this representation of strain:

$$strain^{'}\left( t \right)=\frac{ICP(t)}{V_{rest}\sqrt{{2CA\left( t \right)+C}^{2}P_{ex}^{2}}}$$

Hence, the rate of change of the strain at time t is proportional to the intracycle elastic power moderated by the expression in the denominator.

Maximum Strain versus Tidal Volume and Peak Flow

When ventilating a mechanically heterogeneous system the clinician must choose settings, e.g., mode of ventilation, frequency, tidal volume, inspiratory time, and PEEP that will achieve the ventilation goal while minimizing risk for damage to the patient. In this section we examine the effects of flow for three flow-regulated modes of ventilation (CF, DF, and SF) and tidal volume $\left( V_{T} \right)$ o$n the maximum strain in each of the five compartments$, keeping other ventilator and physiologic parameters constant, i.e., compartmental resistances and compliances, airway resistances, frequency, and PEEP. These parameters are the same as those used in the previous simulations with $PEEP set=2 cm H_{2}O.$With regard to the modes of ventilation, we specify the maximum flow presented by the ventilator. In the cases of constant flow ventilation and decelerating flow ventilation, this will be the initial flow of the ventilator and for sinusoidal flow ventilation, it will be the flow of the ventilator at the inspiratory midpoint. Of course, changes in the tidal volume and maximum flow rate require different inspiratory times $\left( t_{i} \right)$ for each mode of ventilation. For example, in the case of constant flow ventilation:

$V_{T}=Q_{max}t_{i} \Longrightarrow t_{i}=\frac{Q_{max}}{V_{T}} .$

Here are three axes (3D) plots for maximum compartmental strains $\left( S_{max} \right)$ as we vary tidal volume $\left( V_{T} \right)$ and maximum ventilator flow $\left( Q_{max} \right)$. For the other modes of ventilation, the relationship between tidal volume, maximum flow and inspiratory time is a bit more complicated. In the case of constant flow ventilation, the relationship between maximum compartment strain, $S_{max} , a function of both VT and Qmax, can be represented as a surface in 3D space$. We have summarized in the following figure S2A:

**Figure S2 A:** In the heading of each compartment graph, the maximum value of strain over the range of tidal volumes and maximum flows for that compartment is shown. As illustrated, the highest maximum value for strain occurs in Compartment B (2.05938), and the lowest maximum strain value in Compartment E (1.21108). Furthermore, we note the heterogeneity of the maximum strain from compartment to compartment. The same simulations can be performed for decelerating flow (DF) ventilation and sinusoidal flow (SF) ventilation. In the cases of DF and SF ventilation, we detect some nonlinearity in the behavior of the maximum strain as functions of tidal volume and maximum flow. Maximum strain rises more rapidly at higher tidal volumes for a specified maximum flow ($Q_{max}$). This behavior is primarily due to the required increase in inspiratory time, which in turn increases the auto-PEEP, a component of the dynamic strain. Finally, comparing the maximum strains within a given compartment encountered with a given tidal volume, there are variations in the maximum strain according to which mode of ventilation is used:

**Figure S2 B:** Legend as in Figure S1 A. Maximal strain values are specified for each compartment.

**Figure S2 C:** Legend as in Figure S1 A Maximal strain values are specified for each compartment.

Influence of Geometry on Compartmental Stress and Strain

For clarity in this simplified model, we assumed that each compartment was spherical. In actuality, however, geometrical configurations at the microanatomical level assume non-uniform shapes, such as a pyramid, complex polyhedron, or ovoid forms. As a result, the same internal pressures and volume changes create zones of lesser and greater wall stress and strain within their confining boundaries. Examples demonstrating the potential for this regional variation is provided here using ANSYS simulation software (Canonsburg, PA, USA). The inflation of a ‘lung macro-unit’ having ‘compartment D’ (see text figure 2) compliance (.02 L/cmH_2_O) was applied by constant flow inflation over one second to 337 ml tidal volume. The color coding demonstrates areas of maximum (red) and minimum (blue) stress and strain under static end-inspiratory conditions. The distribution of unit stress and strain is not uniform and depends not only upon the baseline geometry (illustrated) but also the stage of inflation (illustrated only for end-inspiration). Such non-uniformity is the result of local variations of stress/strain focusing within the walls of the confining structure. Not illustrated is the local amplification of stress resulting from its interfaces with surrounding lung units.


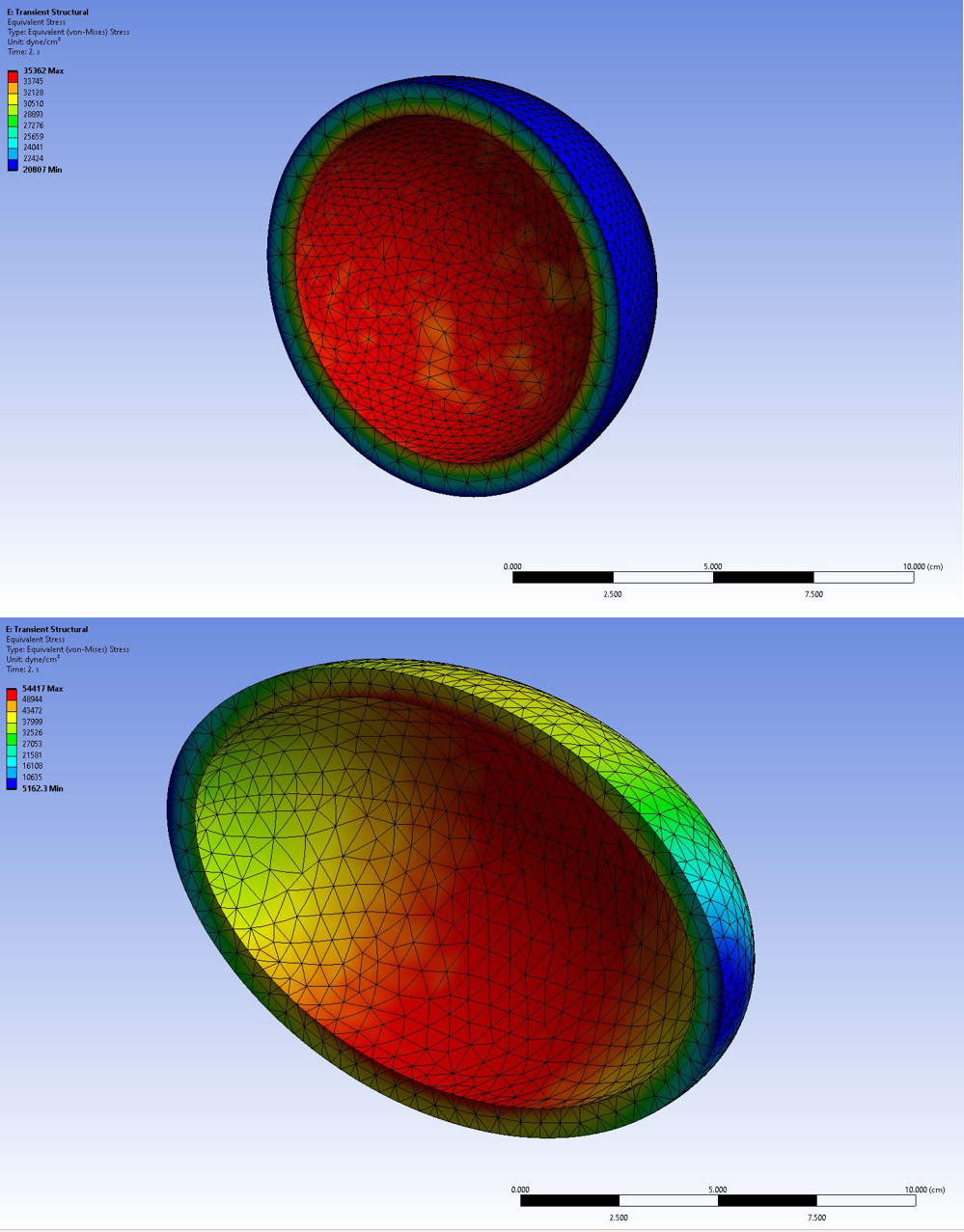


Figure S3 A: An ANSYS simulation demonstrating stress distributions across a spherical (top) and an ovoid (bottom) compartment with a uniform internal pressure. The magnitude and profile of the internal pressure were taken from the mathematical simulations described in this paper and applied to uniform material with characteristics that approximate lung parenchymal tissue.


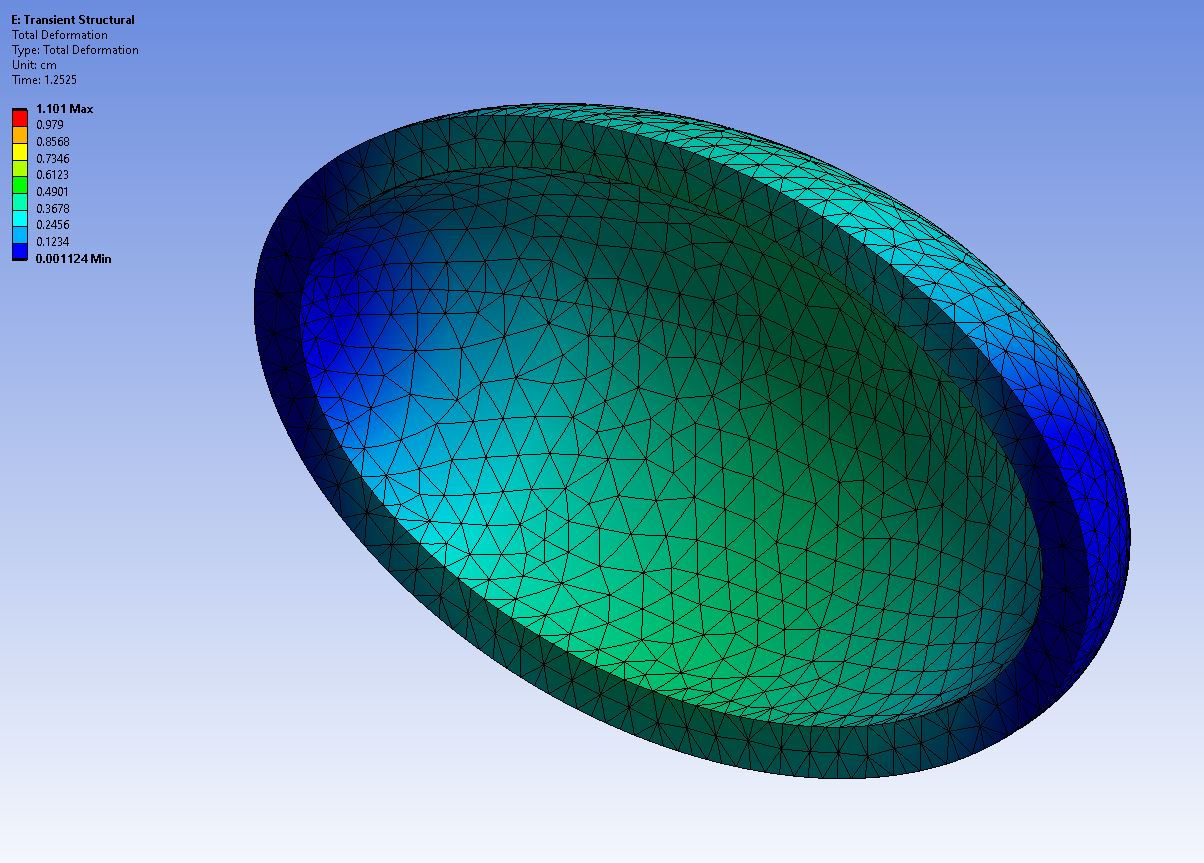

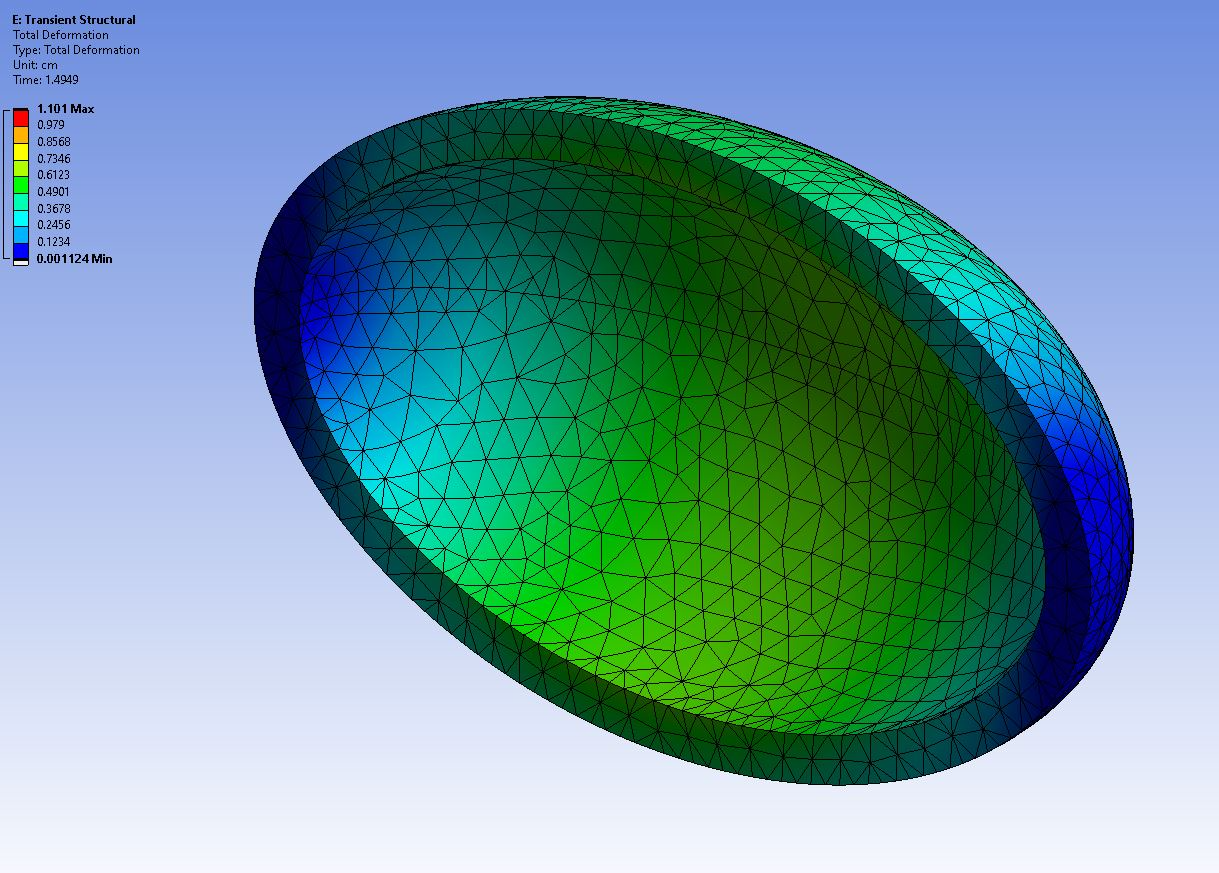


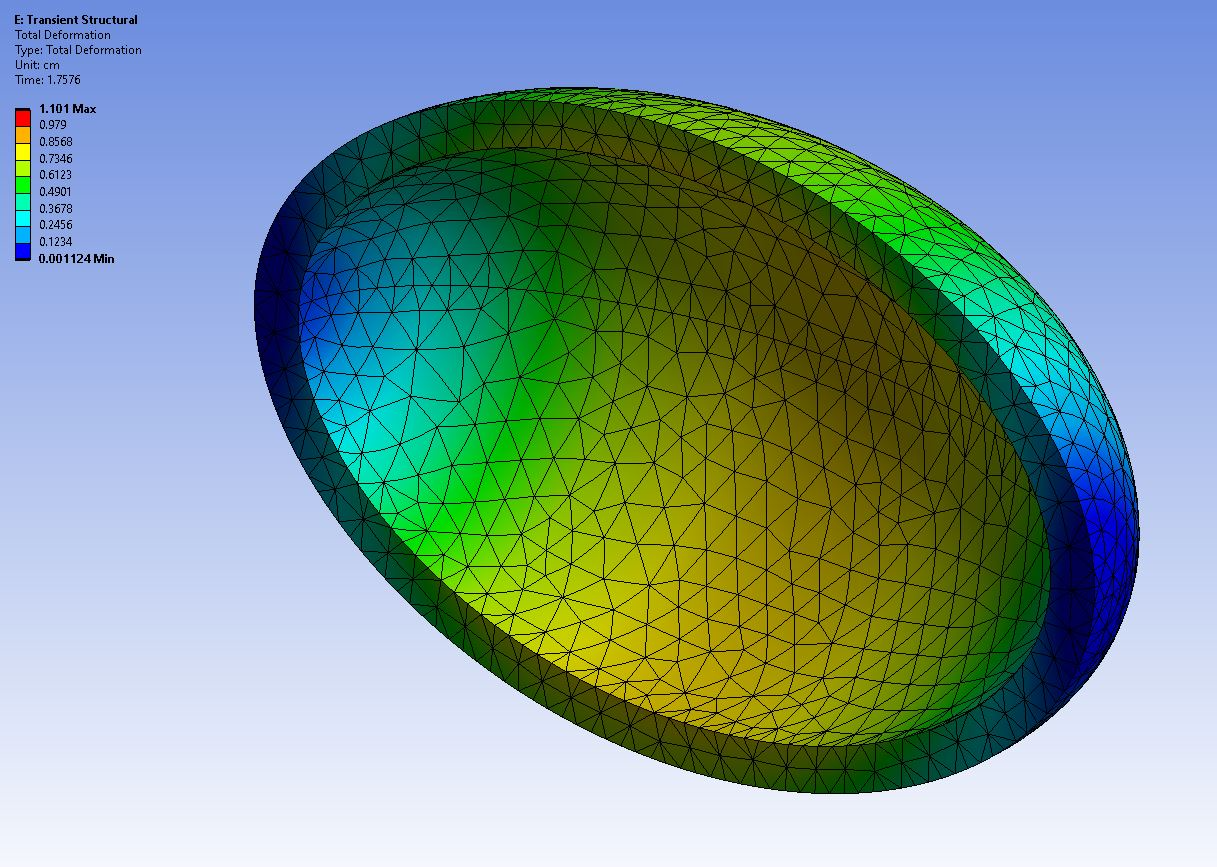

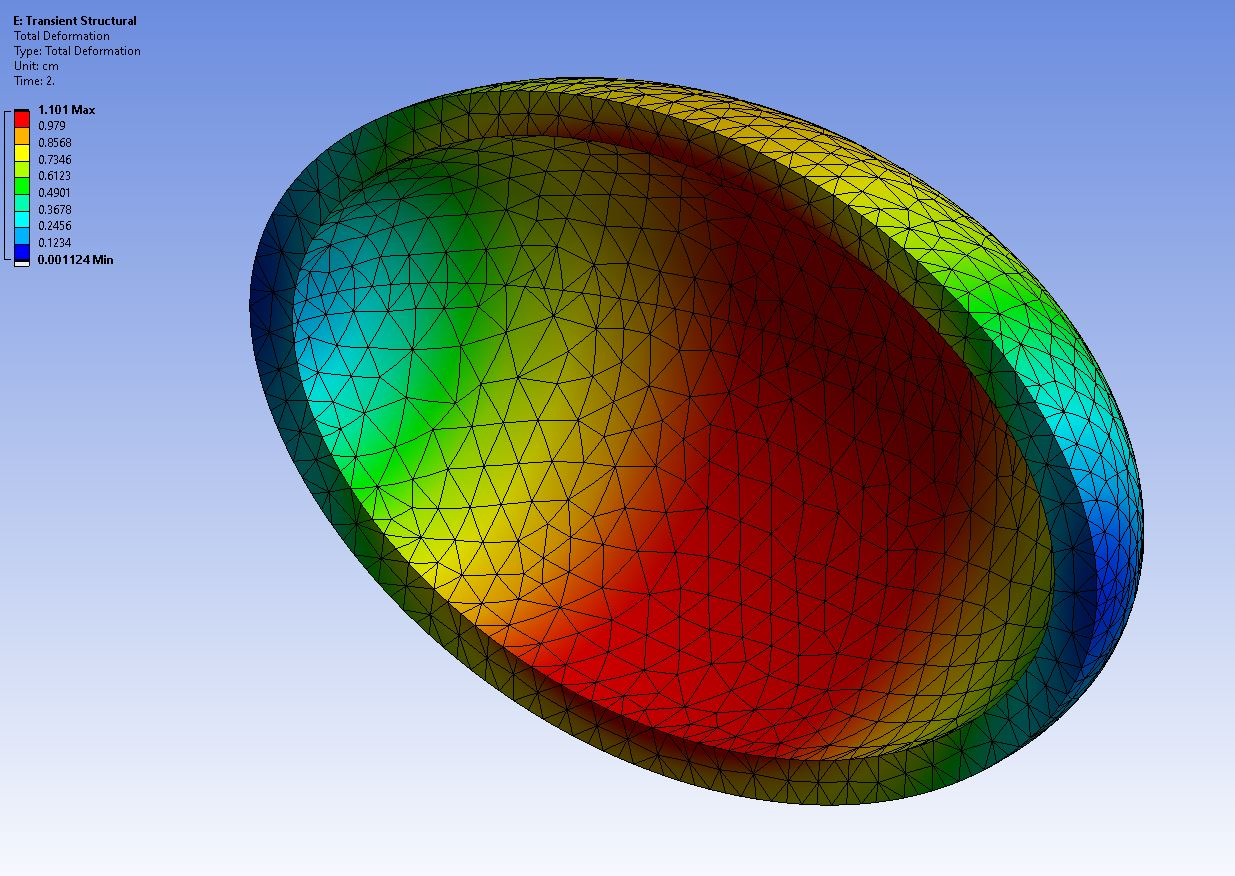


Figure S3 B: An ANSYS simulation demonstrating stress distributions as inflation progresses through the four volume quartiles of a single inspiration (VT) in an ovoid compartment inflated by a uniform internal pressure. Top left: ¼ VT; Top right: ½ VT; Bottom left: ¾ VT; Bottom right: Full VT. Note the regionality of strain magnitudes as inflation proceeds. The magnitude and profile of the internal pressures were taken from the mathematical simulations described in this paper and applied to uniform material with characteristics that approximate lung parenchymal tissue.

**Reference:**

S1) Marini JJ, Crooke PS, Tawfik P, Chatburn RL, Dries DJ, Gattinoni L. Intracycle power and ventilation mode as potential contributors to ventilator‑induced lung injury ICMx (2021) 9:55 <https://doi.org/10.1186/s40635-021-00420-9>.
